# Editorial Comment to Undifferentiated pleomorphic sarcoma of the prostate in a young man

**DOI:** 10.1002/iju5.12182

**Published:** 2020-06-17

**Authors:** Atsuhiko Ochi

**Affiliations:** ^1^ Department of Urology Kameda Medical Center Kamogawa Chiba Japan

Iwahashi *et al*. reported the first experience of diagnosing and treating undifferentiated pleomorphic sarcoma (UPS) of the prostate in a 27‐year‐old man.^1^ UPS is a highly malignant disease and is extremely rare in younger people and in the prostate. Therefore, this is an educative case report that is valuable in finding clues for the treatment of this rare condition.

For metastatic soft tissue sarcoma (STS) chemotherapy, doxorubicin monotherapy or combination therapy with ifosfamide or olaratumab is likely to be used as the first line. Trabectedin, gemcitabine‐docetaxel, and pazopanib are also used in cases of advanced cancer.^2^ However, the prognosis of advanced UPS is poorer than that of other histologic subtypes of STS. Recently, immune checkpoint inhibitors have been receiving increased attention in the treatment of malignant neoplasms. Phase II trials of nivolumab and ipilimumab combination therapy for patients with metastatic STS, including 14% with UPS, reported a confirmed response rate of 16% and overall survival of 14.3 months.^3^ High levels of T‐cell infiltration and PD‐L1 expression were described in UPS compared with that of other histologic subtypes of STS.^4^ It is expected that future research will reveal the efficacy of immune checkpoint inhibitors for the treatment of UPS.

The efficacy of adjuvant/neoadjuvant chemotherapy and radiotherapy focused on histological UPS or prostate sarcomas has not been demonstrated. Therefore, surgery remains the cornerstone of treatment in nonmetastatic UPS. In this report, the tumor recurred at the front of the rectum despite the resected margin having been negative. Moreover, the occurrence of multiple liver metastases is more common in rectal than prostate cancers. I am interested in a potential causal relationship between transrectal biopsy and tumor recurrence in prostate sarcomas. The biopsy tract and scar should be removed at the time of definitive surgery to prevent recurrence from tumor seeding in STS.^5^ If needle biopsy increases the risk of local recurrence, total pelvic exenteration may be recommended in prostate sarcomas diagnosed by transrectal biopsy.

## Conflict of interest

The author declares no conflict of interest.
